# Performance of a Taqman Assay for Improved Detection and Quantification of Human Rhinovirus Viral Load

**DOI:** 10.1038/srep34855

**Published:** 2016-10-10

**Authors:** Kim Tien Ng, Jack Bee Chook, Xiang Yong Oong, Yoke Fun Chan, Kok Gan Chan, Nik Sherina Hanafi, Yong Kek Pang, Adeeba Kamarulzaman, Kok Keng Tee

**Affiliations:** 1 Department of Medicine, Faculty of Medicine, University of Malaya, Kuala Lumpur, Malaysia; 2 Department of Medical Microbiology, Faculty of Medicine, University of Malaya, Kuala Lumpur, Malaysia; 3 Division of Genetics and Molecular Biology, Institute of Biological Sciences, Faculty of Science, University of Malaya, Kuala Lumpur, Malaysia; 4 Department of Primary Care Medicine, Faculty of Medicine, University of Malaya, Kuala Lumpur, Malaysia

## Abstract

Human rhinovirus (HRV) is the major aetiology of respiratory tract infections. HRV viral load assays are available but limitations that affect accurate quantification exist. We developed a one-step Taqman assay using oligonucleotides designed based on a comprehensive list of global HRV sequences. The new oligonucleotides targeting the 5′-UTR region showed high PCR efficiency (*E* = 99.6%, *R*^*2*^ = 0.996), with quantifiable viral load as low as 2 viral copies/μl. Assay evaluation using an External Quality Assessment (EQA) panel yielded a detection rate of 90%. When tested on 315 human enterovirus-positive specimens comprising at least 84 genetically distinct HRV types/serotypes (determined by the *VP4/VP2* gene phylogenetic analysis), the assay detected all HRV species and types, as well as other non-polio enteroviruses. A commercial quantification kit, which failed to detect any of the EQA specimens, produced a detection rate of 13.3% (42/315) among the clinical specimens. Using the improved assay, we showed that HRV sheds in the upper respiratory tract for more than a week following acute infection. We also showed that HRV-C had a significantly higher viral load at 2–7 days after the onset of symptoms (*p* = 0.001). The availability of such assay is important to facilitate disease management, antiviral development, and infection control.

Human rhinovirus (HRV), a member of the *Picornaviridae* family, is a non-enveloped, positive-sense, single stranded RNA virus of approximately 7.2 kb in size. The genome is composed of a 5′-untranslated region (5′-UTR), followed by an open reading frame (ORF) coding for capsid proteins (VP4, VP2, VP3 and VP1), seven non-structural proteins (2A, 2B, 2C, 3A, 3B, 3C and 3D), and a short 3′-UTR poly-A tract. The taxonomic classification of HRV has been recently revised ( http://www.ictvonline.org) with three species of HRV described, namely HRV-A, HRV-B and HRV-C^1^, followed by over 160 genetically distinct types (or serotypes) circulating worldwide.

Although approximately 50% of respiratory tract infections are associated with HRV^2^, they are often considered self-limiting without causing respiratory distress and hence possess little medical significance. However, the advancement in viral detection methods has highlighted the involvement of HRV in lower respiratory compartment leading to severe and possibly fatal respiratory symptoms^3^,^4^, particularly in the elderly, immunocompromised patients^2^ and newborns^5^. In addition, individuals with underlying respiratory diseases such as asthma, chronic bronchitis and cystic fibrosis may have an increased risk of severe HRV-associated complications^6^,^7^.

Several studies have demonstrated a close correlation between the disease severity and HRV viral load in children. For instance, study has shown that a high viral load in respiratory specimens was likely to be associated with lower respiratory tract infection and wheezing in immunocompetent children^8^. Likewise, other study has observed a significant positive correlation between viral load and severity of lower respiratory tract infection such as bronchitis, bronchiolitis and pneumonia, particularly among children^9^. Furthermore, the presence of HRV viremia was associated with a significantly higher nasopharyngeal viral load and more severe disease^10^. Therefore, it is important to readily quantify HRV viral load in assessing disease severity.

Previous studies have attempted to quantify HRV viral load using the real-time PCR approach^10^,^11^,^12^,^13^,^14^,^15^. However limitations that may hinder accurate quantification were evident in many existing assays, such as the lack of sensitivity to detect a broad array of HRV types^11^,^13^ and the potential risk of overestimation of viral load, due to the usage of random hexamer^16^,^17^. To overcome such limitations, we developed an improved real-time reverse transcription-PCR (RT-PCR) assay containing newly designed specific oligonucleotides that correspond to a comprehensive list of global HRV sequences from all HRV species and types. The assay was validated by an HRV external quality assessment (EQA) panel^18^ and tested on HRV-positive nasopharyngeal specimens recently collected from patients presented with symptoms associated with acute respiratory infections. The performance of the assay was compared to that of a commercially available PCR-based HRV quantification kit. Lastly, we estimated the kinetics of HRV viral load in the upper respiratory tract among HRV species, which showed prolonged viral shedding for more than a week during the symptomatic phase of infection. Collectively, the newly developed real-time RT-PCR assay can be used as a detection and quantification tool that may be essential in assessing disease prognosis, monitoring viral suppression during antiviral treatment, and designing infection control measures.

## Results

### HRV detection and species/type determination

**Figure 1:**
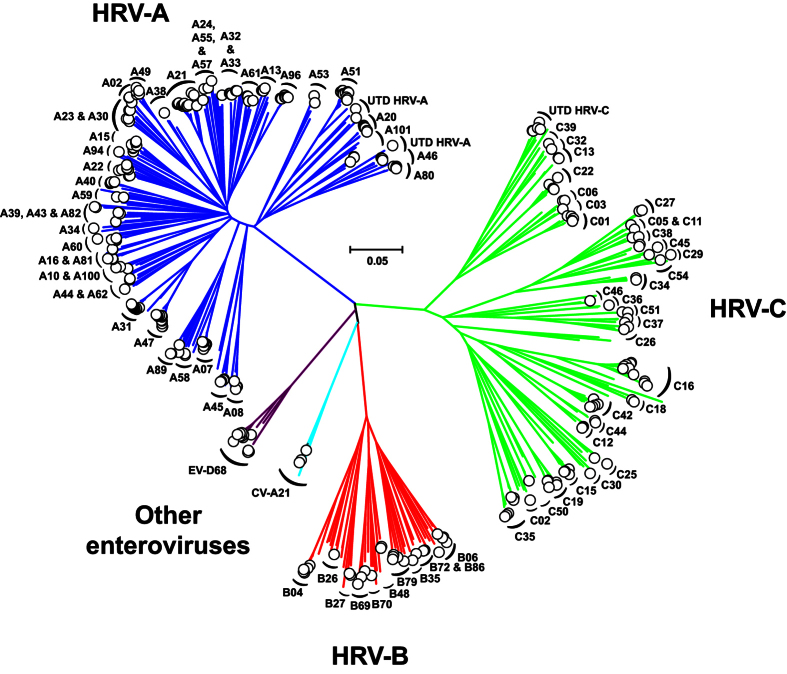
Phylogenetic reconstruction of the *VP4/VP2* gene from 315 HEV-positive nasopharyngeal specimens collected between July 2013 and May 2014 . Neigbour-joining tree analysis indicated that 300 subjects (46.4%) were classified as HRV, of whom 183 (61.0%) were infected with HRV-A, 31 (10.3%) with HRV-B and 86 (28.7%) with HRV-C. HRV types (or serotypes) were indicated at the tips of the tree. In addition, 12 enterovirus D68 and three coxsackievirus A21 sequences were also detected. The statistical significance of the branching order was validated by bootstrap analysis of 1,000 replicates and the scale bar represents 0.05 nucleotide substitutions per site.

Between July 2013 and May 2014, a total of 1,290 consenting outpatients were recruited, of whom 50.1% (646/1290) were positive for at least one viral pathogen in the multiplex respiratory virus panel screening assay. Among 646 subjects, 315 (48.8%) were tested positive for HEV. The *VP4/VP2* gene were successfully amplified and sequenced for all 315 HEV-positive specimens. Phylogenetic analysis showed that 300 subjects (46.4%) were classified as HRV-positive, of whom 183 (61.0%) were infected with HRV-A, 31 (10.3%) with HRV-B and 86 (28.7%) with HRV-C (Fig. 1). The circulating HRV types determined by phylogenetic analysis were as follows: HRV-A21 (15/300, 5.0%), A31 and A47 (12/300, 4.0% each), A07 and C16 (10/300, 3.3% each), A96 (9/300, 3.0%), A20 (8/300, 2.7%), A15, A22 and A32 (7/300, 2.3% each), A13, A16, A49 and C42 (6/300, 2.0% each), A60, A80, B04, B79, C19 and C22 (5/300, 1.7% each), A40, A46, A89, B06, B48 and C01 (4/300, 1.3% each), A02, A23, A61, A101, B69, B72, C15, C34 and C35 (3/300, 1.0% each), A30, A33, A39, A43, A45, A53, A58, A59, A62, A81, A94, A100, B27, C06, C12, C13, C18, C25, C26, C27, C29, C37, C38, C44 and C45 (2/300, 0.7% each), and A10, A24, A34, A38, A44, A55, A57, A82, B26, B35, B70, B86, C02, C03, C05, C11, C30, C32, C36, C39, C46, C50, C51 and C54 (1/300, 0.3% each). In addition, HRV-A and HRV-C types that were not classified with any previously defined types were detected at 1.3% (4/300) and 2.0% (6/300), respectively. In total, 84 distinct HRV types were identified by phylogenetic analysis. In addition, 12 enterovirus D68 and three coxsackievirus A21 sequences were also detected.

### Real-time RT-PCR primers and probe coverage

Primer/probe coverage was defined as the number of alleles in the primers and the probe that complement with the target gene of known/reference sequences. This is a key indicator for improved assay sensitivity^19^ against a broad genetic diversity of HRV species and types. Based on the 384 near complete HRV genomes, the newly designed forward primer, reverse primer and the probe showed a coverage of 95.3%, 95.1% and 97.4% allele identity, respectively, with the global reference sequence alignment. With allowance of 1 mismatch within the oligonucleotides, the coverage improved to 96.6% (forward primer), 96.6% (reverse primer) and 97.4% (probe) (Supplementary Table 1). Based on 1,189 reference sequences (near complete genomes and 5′-UTR), the forward primer, reverse primer and probe revealed a high coverage of 96.9%, 92.0% and 87.6% identity, respectively. The coverage improved to 99.0% (forward primer), 94.2% (reverse primer) and 91.2% (probe) when a single allele mismatch was allowed at least five nucleotides upstream from the 3′ end of the oligonucleotides. Overall, sequence alignment analysis showed a robust and extensive coverage of all HRV species and types by both primers and the probe.

### Performance evaluation of the newly developed real-time RT-PCR assay

**Figure 2:**
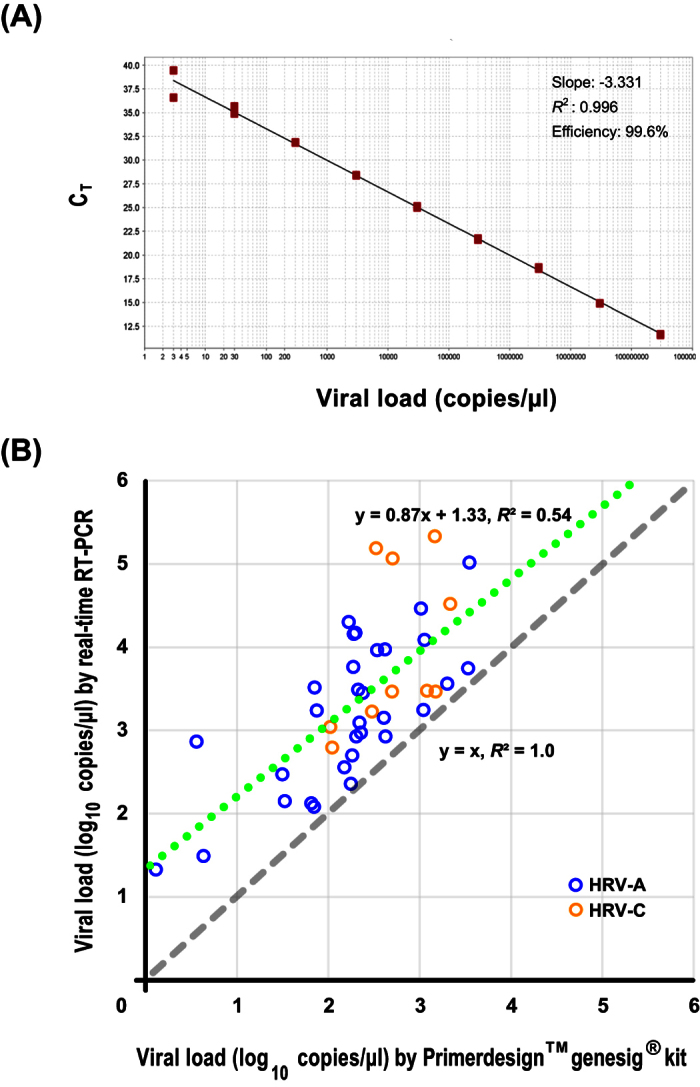
Performance validation and evaluation of the newly developed real-time RT-PCR assay. **(A)** Standard curve of the real-time reverse transcription-PCR assay. In the 10-fold serial dilution of plasmid standards with known concentration (from 3 × 10^0^ to 3 × 10^8^ DNA copies/μl), the slope was estimated at −3.331, with the PCR efficiency (*E*) of 99.6% and Pearson correlation coefficient (*R*^2^) of 0.996. **(B)** Comparison of HRV viral load estimated by real-time RT-PCR and Primerdesign^TM^ genesig^®^ kit based on 40 quantifiable HRV-positive specimens. The dashed diagonal line represents the supposed viral load estimates when the efficiency and accuracy of the real-time RT-PCR assay and the commercial kit are comparable (y = x). The green dotted line (y = 0.87x + 1.33) represents a normalized correlation trendline (*R*^2^ = 0.54) between viral loads estimated by real-time RT-PCR and Primerdesign^TM^ genesig^®^ kit. Specimens are colour-coded according to the HRV species (HRV-A, blue; HRV-C, tangerine).

When the real-time RT-PCR assay was performed on serially diluted plasmid (Clone 04 that contains 5′-GGCCCCTGAATGTGCTAA-3′ and 5′-ATGGGACCAACTACTTTG-3′ sequences) standards of known concentration, the PCR efficiency of the newly developed assay was estimated at 99.6%, with a coefficient of determination (*R*^2^) of 0.996 (Fig. 2A). When evaluated using the EQA panel, the newly developed assay detected 9 out of 10 core and educational samples, producing a success rate of 90% (Table 1). The variability (expressed in coefficient of variation, CV) was studied by analysing EQA panel tested in triplicate. The results revealed that the intra- and inter-assay CV were 0.85% and 4.20%, respectively. The educational sample of HRV-A16, which was reported as “infrequently detected” by QCMD, was not detected by the assay. The C_T_ values recorded by the assay were largely similar to that of the panel recommended by QCMD. PCR amplification of the *VP4/VP2* gene from the EQA panel showed all but one (the educational HRV-A16 sample) were amplified successfully. When tested on HEV-positive clinical specimens, the assay detected all HRV of different species and types (300/300, 100%) as well as the enterovirus D68 (12/12, 100%) and coxsackievirus A21 (3/3, 100%) specimens (Table 2). However, only 290/300 (96.7%) were quantifiable (range: 2–275,422 viral copies/μl), whereas the remaining 10 samples were <1 viral copy/μl. The assay turnaround time was approximately 45 minutes.

To evaluate the effects of primers and probe mismatches on HRV quantification efficiency, a total of 16 plasmids (that contain all 16 combinations of qR447f and R529 sequences) were constructed and evaluated in triplicates (Supplementary Table 2), in which, the C_T_ values, PCR efficiency (*E*), coefficient of determination (*R*^*2*^) and dynamic range were largely similar (Supplementary Figure 1 and Supplementary Table 3). This suggests that the used of degenerate oligonucleotides in present study poses minimum impact on the annealing kinetics.

When the commercially available Primerdesign^TM^ genesig^®^ Human Rhinovirus All Subtypes (generic) kit was assessed, the RT-PCR efficiency of the kit was estimated at 96.5%, with an *R*^2^ of 0.998. When evaluated using the EQA panel, the kit was unable to detect HRV in all 10 core and educational samples (Table 1). Evaluation using the HEV-positive clinical specimens revealed a very low detection rate of 13.3% (42/315), in which only some HRV-A (32/183) and HRV-C (10/86) specimens were detected. More specifically, the detected HRV types include HRV-A02, A07, A20, A22, A30, A32, A55, A58, A60, A61, A80, A94, A100, C01, C22 and C44. None of the HRV-B, enterovirus D68 and coxsackievirus A21 samples were detected (Table 2). Of the 42 clinical specimens, 40 (95.2%) were quantifiable (range: 3–3,467 viral copies/μl), with two specimens estimated at <1 viral copy/μl. The turnaround time for the commercial kit was approximately 90 minutes. When the viral load from a single individual generated by the two assays were compared, it was shown that the viral load estimated by the commercial kit was lower than those determined by the real-time RT-PCR in all individuals (Fig. 2B), suggesting the potential risk of viral load underestimation by the commercial kit.

### Viral load estimation between HRV species during acute phase of infection

**Figure 3:**
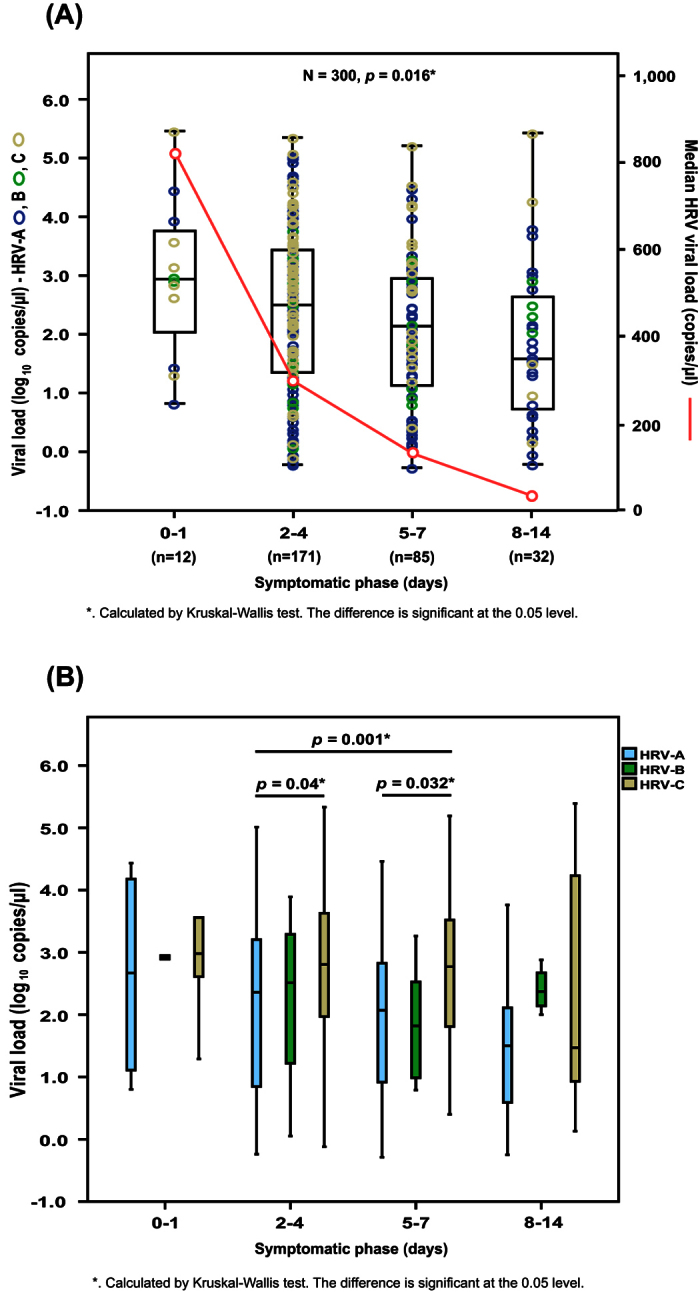
The dynamicity of population viral load in the upper respiratory tract during symptomatic phase of acute HRV infection. **(A)** Boxplot illustrating the changes of HRV viral load (in log_10_ copies/μl) during the course of acute respiratory tract infection. The regressive trendline highlighted a significant difference in median viral load (in copies/μl) between the estimated number of days from onset of symptoms (*p* = 0.016). **(B)** Boxplot illustrating the HRV species and their viral loads (in log10 copies/μl) during the symptomatic phase of acute HRV infection. The comparison of viral load between HRV species indicated that HRV-C had a significantly higher viral load than HRV-A and HRV-B, particularly at 2–7 days (*p* = 0.001). The difference in viral load between HRV-A and HRV-B was not statistically significant. The HRV-positive specimens are colour-coded, based on the HRV species. The *p*-values were calculated by Kruskal-Wallis test and the difference is significant at the 0.05 level.

To investigate the kinetics of HRV population viral load during the symptomatic phase of infection, cross sectional viral load was measured from 300 subjects for up to two weeks. The data were analyzed through linear correlation (bivariate and partial) as well as the non-parametric Mann-Whitney U test and Kruskal-Wallis test using the Statistical Package for Social Sciences version 22.0 (SPSS Inc., Chicago, USA). A significant negative correlation between HRV viral load and the estimated number of days from onset of symptoms was observed, with a correlation coefficient (*r*) of −0.174 (*p* = 0.003). A significant regression in median HRV viral load (*p* = 0.016) was observed; from 827 viral copies/μl at 0–1 day to 298 viral copies/μl at 2–4 day and 132 viral copies/μl at 5–7 day, before declining to <50 viral copies/μl at 8–14 day (Fig. 3A). Of note, all subjects with respiratory symptoms for 8–14 days had detectable HRV. At the species level, it was shown that HRV-C had a significantly higher log viral load as compared to HRV-A and HRV-B at 2–4 (*p* = 0.04) and 5–7 day (*p* = 0.032) (Fig. 3B). The difference in viral load between HRV-A and HRV-B however was not statistically significant.

## Discussion

Acute respiratory infection is the most common infectious disease in human population. In comparison to other viruses that have been frequently implicated to cause acute respiratory infection such as the influenza viruses^20^,^21^, HRV are often considered innocuous, although nearly half of all respiratory tract infections have been associated with HRV^2^. However, increased surveillance and the advancement in viral detection methods have established the involvement of HRV in respiratory tract infections that in some cases could lead to severe and possibly fatal respiratory symptoms^3^,^5^,^22^,^23^.

In the attempt to identify factors associated with disease severity, several studies have demonstrated a close correlation between the HRV viral load and severity of acute respiratory illnesses, particularly among children^8^,^9^ and those with underlying predisposing respiratory conditions^22^,^23^,^24^. Other studies however have shown contradictory findings, where no correlation between viral load and disease severity was observed^25^,^26^. Although the use of real-time RT-PCR approach for HRV viral load quantification is common, a number of limitations that may result in inaccurate quantification continue to exist. To tackle these challenges, we developed a one-step real-time RT-PCR assay using specific primers and probe designed based on an updated and comprehensive list of reference sequences. The efficiency of the newly designed assay was estimated from a standard curve plotted using a serially diluted plasmid containing the HRV 5′-UTR region. A constructed plot of the C_T_ values versus the logarithm of the target concentrations is expected to be linear with a negative slope (−3.33 for a 10-fold dilution series) when the assay efficiency (*E*) is 100%^27^. In addition, the plot should also yield a Pearson correlation coefficient (*R*^2^) of greater than 0.97^28^. In our real-time RT-PCR, with an estimated *E* = 99.6%, *R*^2^ = 0.996 and a slope of −3.33 (Fig. 2A), the newly designed reagents and protocol are considered efficient and robust. In addition, its performance was further evaluated using the quality assessment panel that yielded a detection rate of 90%, of which one of the educational samples was not captured. PCR amplification of HRV *VP4/VP2* gene from this sample also yielded a negative result, suggesting possible very low copy number or absence of HRV in the sample.

Since the EQA panel consists only a minor fraction of HRV types (HRV-A8, -A16, -A90, -B5 and -C), we went on to investigate the performance of the newly designed assay using clinical specimens, viral load from 315 HEV-positive nasopharyngeal swabs were measured using the assay. Of the 315 HEV-positive nasopharyngeal swabs, HRV-A was predominantly detected, infecting over 60% of the subjects, followed by HRV-C, HRV-B and other enteroviruses. Remarkably, the assay yielded a 100% (315/315) success rate, detecting all HRV strains of different species (and types) as well as enterovirus D68 and coxsackievirus A21 (Table 1). In light of the recent enterovirus D68 outbreaks in various parts of the world^29^, the ability of the newly developed assay to quantify enterovirus D68 viral load in clinical specimens may be of additional value. When compared to the commercially available kit, the newly designed assay, which has a shorter turnaround time, was more robust with improved sensitivity in detecting and quantifying a broad array of HRV types in clinical nasopharyngeal specimens (Fig. 2B). In addition, due to its ability to detect other non-HRV enteroviruses, we went on to test the assay on a collection of archived specimens (collected between 1997 and 2013) of diverse non-polio enteroviruses that have been associated with various human diseases. Interestingly, the assay was able to detect and quantify these specimens, which consist of human enterovirus A71 (n = 9), coxsackieviruses (A6, A16, and B1–B5) (n = 29), and echoviruses (E6, E7, E11, and E19) (n = 6), yielding a success rate of 100% (viral load data not shown). The expanded coverage beyond HRV clearly demonstrated the wide applicability of the assay on various enteroviruses, although a more comprehensive evaluation may be necessary.

Given the extensive genetic diversity and the growing evidence that associates HRV with more severe lower respiratory tract infections, the development of an improved real-time RT-PCR for HRV detection and quantification is relatively timely, highlighting its clinical importance and potential application as a diagnostic tool for respiratory illnesses. The increased sensitivity and accuracy in estimating HRV viral load is essential especially in the attempt to establish the impact of HRV viral load on the risk of more severe lower respiratory tract complications such as the chronic obstructive pulmonary disease (COPD) and asthma exacerbation^30^. Furthermore, such assay may also be useful in monitoring HRV viral load in clinical trials of potential antiviral candidates e.g. vapendavir or pleconaril^31^, thus determining the effectiveness of the treatment.

Likewise, the availability of the improved quantification assay enables better understanding of HRV pathogenesis through the duration of viral shedding during acute respiratory infections. Previous study revealed that following a 2-day incubation period^32^, HRV sheds for approximately 10 days in the upper respiratory compartment following acute infection, of which the duration of respiratory tracts symptoms correlates with the duration of virus shedding^33^. Since 25 out of 32 of the study subjects had detectable viral load 14 days after the onset of symptoms (Fig. 3A), our data suggest that HRV could potentially shed in the respiratory tract of some individuals longer than previously reported, implying that viral transmission could still occur during the second week of infection. Therefore, optimized infection control measures as well as the course of treatment should be implemented and extended accordingly to minimize onward transmission of HRV during this period. However, since several HRV types can circulate simultaneously in the population, sequential or mixed infections by another HRV strain(s) following primary infection are not uncommon^34^, contributing to an increase in viral load. Nevertheless, the prolonged shedding of HRV viral load observed in this cohort should warrants further investigation especially among a larger study population. Lastly, our data revealed that HRV-C had a significantly higher viral load than HRV-A and -B, particularly during the 2–7 days after the onset of symptoms (Fig. 3B). This probably explains the increased disease severity attributed to HRV-C as compared to HRV-A and -B infection as recently reportedly elsewhere^35^,^36^. However, such association between HRV species, viral load and the severity of respiratory illness remains debatable^10^,^24^ unless a more comprehensive study involving a more in-depth sampling method spanning a longer recruitment period is conducted.

In summary, we have developed an improved real-time RT-PCR assay for HRV detection and quantification. The assay was validated using an external quality assurance panel of HRV and tested on more than 300 clinical specimens representing at least 84 genetically distinct HRV types, in which the newly developed assay performance was more superior than a commercially available kit. We went on to show that prolonged viral shedding in the upper respiratory tract for more than a week following acute infection was common.

## Materials and Methods

### Ethics Statement

This study was approved by the University Malaya Medical Centre Medical Ethics Committee (MEC890.1). Standard, multilingual consent forms validated by the Medical Ethics Committee were used. Written informed consent was obtained from all subjects prior to sample collection. All experiments were performed in accordance with approved guidelines and regulations.

### Study Subjects and Specimens

In the present study, a total of 1,290 consenting outpatients (age range: 13–95 years old) who presented with symptoms of acute upper respiratory tract infections were recruited at the Primary Care Clinic, University Malaya Medical Centre, Kuala Lumpur, Malaysia between July 2013 and May 2014. Respiratory specimens in the form of nasopharyngeal swabs were collected in universal transport medium using standardised method. The presence of symptoms associated with acute respiratory tract infection (sneezing, nasal discharge, nasal obstruction, headache, sore throat, hoarseness of voice, muscle ache and cough) was determined based on previously published criteria^37^,^38^ and the number of days after the onset of symptoms (symptomatic phase) was recorded.

### Detection and Identification of Human Rhinovirus

Total viral RNA was extracted from nasopharyngeal specimens using the NucliSENS easyMAG automated nucleic acid extraction system (bioMérieux, Marcy I’Etoile, France)^39^, as described in the manufacturer’s protocol. The specimens were screened for viral pathogens using the xTAG Respiratory Viral Panel (RVP) FAST Assay (Luminex Molecular, Toronto, Canada) and analysed using the Luminex’s proprietary Universal Tag sorting system on the Luminex 200 IS platform (Luminex Corp, Texas, USA)^40^. The respiratory virus panel comprises of the human enteroviruses (HEV) (including HRV and other enteroviruses), influenza A virus, influenza B virus, respiratory syncytial virus, human coronaviruses, parainfluenza viruses, human metapneumovirus, human bocavirus, and adenovirus.

Specific enteroviruses in HEV-positive specimens were further confirmed using standard molecular approach that involved amplification and direct sequencing of the *VP4/VP2* gene (549 bp) using primers described previously^41^. To deduce viral phylogeny and determine the genetic type of HEV, neighbour-joining tree was reconstructed based on the *VP4/VP2* gene using MEGA version 6.0 based on Kimura 2-parameter model^42^. The statistical robustness of the branching orders was assessed by bootstrap analysis of 1,000 replicates.

### Design and Development of Real-Time Reverse Transcription-PCR (RT-PCR)

The new primers and probe for one-step real-time RT-PCR targeting the highly-conserved 5′-UTR region of HRV were designed based on 1,189 reference sequences (384 near complete genome and 805 5′-UTR region from all HRV types) retrieved from the GenBank (accessed on 24 October 2014). The newly designed primers are qR447f (5′-GGCCCCTGAATGYGGCTAA-3′, nucleotide position relative to human rhinovirus 1B [accession number D00239.1]: 450–468) and qR561r (5′-GAAACACGGACACCCAAAGTAG-3′, nucleotide position: 542–564), with the FAM-labeled probe R529 (5′-AYGGRACCRACTACTTTG-3′ (nucleotide position: 532–550).

These primers and probe were designed using Primer Express (Applied Biosystems, California, USA) with a melting temperature between 65–66.5 °C. In addition, the high hairpin ΔG (−1.39 kcal/mole) and self-dimer ΔG (−9.28 kcal/mole), suggesting a low potential of secondary structure and self-dimer formation. To evaluate the effects of primers and probe mismatches on HRV quantification efficiency, 16 HRV 5′-UTR that represent all 16 combinations of qR447f and R529 sequences were amplified, cloned into plasmid using pTOPO-TA cloning (Invitrogen, California, USA) and evaluated in triplicates. A standard curve of the cycle threshold (C_T_) values obtained from serial dilutions of the plasmid (3 × 10^0^ to 3 × 10^8^ copies/μl) was constructed, where the C_T_ values of the unknown samples were plotted on the standard curve for viral load determination. The plasmid containing qR447f (5′-GGCCCCTGAATGTGGCTAA-3′) and R529 (5′-ATGGGACCAACTACTTTG-3′) sequences that covers the most HRV types was used to construct a standard curve of C_T_. The viral load was expressed in viral copies/μl of extracted RNA.

To evaluate the performance of the newly developed assay, viral load of HEV-positive clinical specimens were determined by adding 3 μl of extracted viral RNA to a 15 μl reaction mixture containing 10 μM of each primer, 10 μM of probe, 2x SensiFAST probe (Lo-Rox) one-step mix and reverse transcriptase (Bioline, London, UK). The real-time amplification was performed on ViiA 7 Real-time PCR system (Applied Biosystems, USA) with the thermal cycling profile as follows: 8 minutes of reverse transcription at 48 °C, 2 minutes of polymerase activation at 95 °C, followed by 42 cycles of denaturation (95 °C for 5 seconds) and annealing (60 °C for 20 seconds). Negative controls with RNAse-free water were included to check for carry-over contamination.

### Evaluation of the Real-time RT-PCR Assay using External Quality Assessment (EQA) Panel and Primerdesign^TM^ genesig^®^ Kit

A 2013 rhinovirus external quality assessment (EQA) panel prepared in collaboration with Quality Control for Molecular Diagnostics (QCMD) was used to evaluate the performance of the newly designed assay^18^. The HRV species/types included in the EQA panel are HRV-A8, A16, A90, HRV-B5 and HRV-C prepared in transporting medium with different dilution factors (concentration in copies/ml), ranges from 10^−6.5^ to 10^−2^. In addition, a sample of enterovirus D68 was included in the panel. The panel consists of two sample types, namely the core and educational samples. Briefly, the sample types correspond to the expected detection rate of the panel, in which the core samples are those commonly detected (95%) in routine testing, whereas the educational samples are samples with either low virus titre or contain new circulating strains that may be infrequently detected. In addition to the only negative control from EQA panel, three other negative controls with RNAse-free water were included in each batch of experiments.

To assess the performance of the newly developed assay, viral load estimation was compared with a commercially available Primerdesign^TM^ genesig^®^ Human Rhinovirus All Subtypes (generic) kit for *in vitro* quantification (Catalogue number Path-HRVsp-standard and OneStep-oasig150) using the EQA panel as well as the HEV-positive clinical specimens, performed on a ViiA 7 Real-time PCR system, in accordance to the manufacturer’s protocol. According to the product description, the kit targets the 5′-UTR region of HRV and has a broad dynamic detection range (>6 logs) with high sensitivity to detect specimens with less than 100 copies of viral RNA.

## Additional Information

**How to cite this article**: Ng, K. T. *et al*. Performance of a Taqman Assay for Improved Detection and Quantification of Human Rhinovirus Viral Load. *Sci. Rep.* **6**, 34855; doi: 10.1038/srep34855 (2016).

## Supplementary Material

Supplementary Information

## Figures and Tables

**Table 1:** Performance of the newly developed real-time RT-PCR assay and the commercially available kit against the external quality assessment (EQA) panel recommended by the Quality Control for Molecular Diagnostics (QCMD).

External Quality Assessment (EQA) panel, Quality Control for Molecular Diagnostics (QCMD)				In-house real-time RT-PCR assay		Primerdesign^TM^ genesig^®^ Kit		Amplification of *VP4/VP2* gene
HEV species	QCMD classification*	Genetic type#	C_T_ value#	Detection	C_T_ value	Detection	C_T_ value	
HRV-A	Core	A08	42.94	+	39.09	−	−	+
	Core	A16	35.27	+	36.82	−	−	+
	Core	A90	39.11	+	39.14	−	−	+
	Educational	A16	41.67	−	−	−	−	−
	Educational	A90	44.33	+	39.05	−	−	+
HRV-B	Core	B05	27.79	+	29.77	−	−	+
	Educational	B05	34.53	+	35.63	−	−	+
HRV-C	Core	N/A	30.84	+	30.19	−	−	+
	Educational	N/A	35.58	+	33.27	−	−	+
Enterovirus D68	Educational	N/A	N/A	+	38.87	−	−	+
Negative control	Core	N/A	N/A	−	−	−	−	−
Overall performance				9/10 (90%)		0/10 (0%)		9/10 (90%)

^*^The classification corresponds to the expected detection rate of the panel, in which the cores are those commonly detected (95%) in routine testing, whereas the educational samples refer to samples with either low titre or contain new circulating strains.

^#^Obtained from Quality Control for Molecular Diagnostics (QCMD) RVRNA13 External Quality Assessment (EQA) final report.

N/A, Not available.

**Table 2:** Performance of the newly developed real-time RT-PCR assay and the commercially available kit against 315 human enterovirus-positive nasopharyngeal specimens collected between July 2013 and May 2014.

HEV species from clinical specimens (nasopharyngeal swab), n = 315	In-house real-time RT-PCR assay	Primerdesign^TM^ genesig^®^ Kit	Amplification of *VP4/VP2* gene
HRV-A (n = 183)	183 (100%)	32 (17.5%)	183 (100%)
HRV-B (n = 31)	31 (100%)	0 (0.0%)	31 (100%)
HRV-C (n = 86)	86 (100%)	10 (11.6%)	86 (100%)
Enterovirus D68 (n = 12)	12 (100%)	0 (0.0%)	12 (100%)
Coxsackievirus A21 (n = 3)	3 (100%)	0 (0.0%)	3 (100%)
Overall performance	315/315 (100%)	42/315 (13.3%)	315/315 (100%)
