# Supplementary material for: Performance of a Taqman Assay for Improved Detection and Quantification of Human Rhinovirus Viral Load
Source: Sci Rep. 2016 Oct 10;6:34855. doi: 10.1038/srep34855 (PMC5056400; doi:10.1038/srep34855)
Supplement: Supplementary Information [file srep34855-s1.pdf]

Supplementary Table 1

Performance of a Taqman Assay for Improved Detection and Quantification of Human Rhinovirus Viral Load

Kim Tien Ng, Jack Bee Chook, Xiang Yong Oong, Yoke Fun Chan, Kok Gan Chan, Nik Sherina Hanafi, Yong Kek Pang, Adeeba Kamarulzaman, and Kok Keng Tee

Coverage of primer sets and probe in 384 HRV near complete genomes

	Name	Primer Sequence	%Coverage (N = 384)	
			100% identity	One mismatch
This study	qR447f	GGCCCCTGAATGYGGCTAA	95.3	96.6
	qR561r	GAAACACGGACACCCAAAGTAG	95.1	96.6
	R529pf	AYGGRACCRACTACTTTG	97.4	97.4
Other studies	Forward 1	WGCCYGCGTGGCKGCC	87.2	87.8
	Reverse	GAAACACGGACACCCAAAGTAGT	95.1	96.6
	Probe	CTCCGGCCCCTGAATGYGGCTAA	94.3	96.4
	Pic-1	TCCTCCGGCCCCTGAAT	0.26	94.0
	Pic-3	GAAACACGGACACCCAAAGTAGT	95.1	96.6
	Pic-5	YGGCTAACCYWAACCC	61.2	72.4
	Forward	CGGCCCCTGAATGCGGCTAA	44.3	95.3
	Reverse	GAAACACGGACACCAAAGZTA	0.00	0.00
	Probe	TCTGCAGCGGAACCGACTA	0.00	0.00
	RV16TMF	CGCTCAGCTGTTAACCCAACA	1.04	1.56
	RV16TMR	CAGCCACGCAGGCTAGAAC	34.6	43.5
	RV16TMP	TAGAGATTCCCCTCCGGCGACGG	1.3	2.1
	Forward	AGCCTGCGTGGCKGCC	79.7	87.8
	Reverse	GAAACACGGACACCCAAAGTAGT	95.1	96.6
	Probe	CTCCGGCCCCTGAATGYGGCTAA	94.3	96.4

References:

1 Dupouey, J. *et al.* Molecular detection of human rhinoviruses in respiratory samples: a comparison of Taqman probe-, SYBR green I- and BOXTO-based real-time PCR assays. *Virol J* **11**, 31, doi:10.1186/1743-422X-11-31 (2014).

2 Do, D. H. *et al.* A one-step, real-time PCR assay for rapid detection of rhinovirus. *J Mol Diagn* **12**, 102-108, doi:10.2353/jmoldx.2010.090071 (2010).

3 Kares, S. *et al.* Real-time PCR for rapid diagnosis of entero- and rhinovirus infections using LightCycler. *J Clin Virol* **29**, 99-104 (2004).

4 Lu, X. *et al.* Real-time reverse transcription-PCR assay for comprehensive detection of human rhinoviruses. *J Clin Microbiol* **46**, 533-539, doi:10.1128/JCM.01739-07 (2008).

5 Sachs, L. A., Schnurr, D., Yagi, S., Lachowicz-Scroggins, M. E. & Widdicombe, J. H. Quantitative real-time PCR for rhinovirus, and its use in determining the relationship between TCID50 and the number of viral particles. *J Virol Methods* **171**, 212-218, doi:10.1016/j.jviromet.2010.10.027 (2011).

6 Esposito, S. *et al.* Impact of rhinovirus nasopharyngeal viral load and viremia on severity of respiratory infections in children. *Eur J Clin Microbiol Infect Dis* **33**, 41-48, doi:10.1007/s10096-013-1926-5 (2014).

7 Schibler, M. *et al.* Experimental human rhinovirus and enterovirus interspecies recombination. *J Gen Virol* **93**, 93-101, doi:10.1099/vir.0.035808-0 (2012).

8 Kotla, S., Major, S. C. & Gustin, K. E. Rapid detection and quantitation of poliovirus and rhinovirus sequences in viral stocks and infected cells. *J Virol Methods* **157**, 32-39, doi:10.1016/j.jviromet.2008.12.005 (2009).

Supplementary Table 2

Performance of a Taqman Assay for Improved Detection and Quantification of Human Rhinovirus Viral Load

Kim Tien Ng, Jack Bee Chook, Xiang Yong Oong, Yoke Fun Chan, Kok Gan Chan, Nik Sherina Hanafi, Yong Kek Pang, Adeeba Kamarulzaman, and Kok Keng Tee

The identity of plasmid constructs containing specific variant (underlined) sequences

Clone	qR447f (5′ to 3′)	Sequence between qR447 and R529 (5′ to 3′)	R529 (5′ to 3′)
01	GGCCCCTGAATGTGGCTAA	TCCTAACCCCGCAGCTATCGTACGCAATCCAGCGTATTGGTAGTCGTAATGAGCAATTGTGGG	ATGGAACCGACTACTTTG
02	GGCCCCTGAATGCGGCTAA	CCTCAACCCCGGAGCCTTGTATTGCAATCCAGCAATATTAAGGTCGTAATGAGCAATTCTGGG	ATGGGACCGACTACTTTG
03	GGCCCCTGAATGTGGCTAA	CCTTAACCCTGCAGCTAGAGCATACAAACCAGTGTGTATCTAGTCGTAATGAGCAATTGCGGG	ACGGGACCGACTACTTTG
04	GGCCCCTGAATGTGGCTAA	TCCTAACCCCGCAGCTGTAGTGTGCAATCCAGCATATTTGCAGTCGTAATGGGTAACTGCGGG	ATGGGACCAACTACTTTG
05	GGCCCCTGAATGCGGCTAA	CCTTAACCCTGCAGCTAGTGCATACAATCCAGTGTGTGGCTAGTCGTAATGAGCAATTGCGGG	ATGGGACCAACTACTTTG
06	GGCCCCTGAATGTGGCTAA	CCTTAAACCTGCAGCCATGGCTCATAAACCAATGAGTTTGTGGTCGTAATGAGTAATTGCGGG	ATGGGACCGACTACTTTG
07	GGCCCCTGAATGTGGCTAA	CTCCAACCCCACAGCCAATGCATGTAAACCAACATGTATTTGGTCGTAACGGGTAACTGTGGG	ACGGAACCGACTACTTTG
08	GGCCCCTGAATGTGGCTAA	TCCTAACCCCGTAGCTGTTGCATGCAACCCAGCATGTATGCAGTCGTAATGGGCAACTATGGG	ATGGAACCAACTACTTTG
09	GGCCCCTGAATGTGGCTAA	CCTTAACCCTGCAGCTAGTGCATACAAGCCAGTATGTAGCTAGTCGTAATGAGCAATTGCGGG	ACGGGACCAACTACTTTG
10	GGCCCCTGAATGTGGCTAA	CTCCAACCCTGCAGCCATTGCATGGAAGCCACCATGTGGATGGTCGTAATGAGTAATTGCGGG	ACGGAACCAACTACTTTG
11	GGCCCCTGAATGCGGCTAA	CCTTAACCCTGCAGCCATTGCCTACAAGCCAGTGGGTATGTGGTCGTAATGAGAAATTGCAGG	ACGGGACCGACTACTTTG
12	GGCCCCTGAATGCGGCTAA	CTCCAACCCCACAGCTATGGCATGCAATCCAGCATGTGCGTAGTCGTAATGAGTAATTGTGGG	ACGGAACCGACTACTTTG
13	GGCCCCTGAATGCGGCTAA	TCCTAACCCCGCAGCTGTAGTGTGCAATCCAGCATATTTGCAGTCGTAATGGGTAACTGCGGG	ATGGAACCAACTACTTTG
14	GGCCCCTGAATGCGGCTAA	CCTTAACCCTGCAGCTAGTGCATGTAATCCAACATGTTGCTAGTCGTAATGAGCAATTGCGGG	ACGGGACCAACTACTTTG
15	GGCCCCTGAATGCGGCTAA	CCTTAACCCCGGAGCCCAGTGACATAATCCAATGTTATTTGGGTCGTAATGAGTAATTCCGGG	ATGGAACCGACTACTTTG
16	GGCCCCTGAATGCGGCTAA	CCTTAACCCTGCAGCTAGTACATGCAATCCAGCATGTTGCTAGTCGTAACGAGCAATTGCGGG	ACGGAACCAACTACTTTG

Supplementary Figure 1

Performance of a Taqman Assay for Improved Detection and Quantification of Human Rhinovirus Viral Load

Kim Tien Ng, Jack Bee Chook, Xiang Yong Oong, Yoke Fun Chan, Kok Gan Chan, Nik Sherina Hanafi, Yong Kek Pang, Adeeba Kamarulzaman, and Kok Keng Tee

Standard curves of the real-time reverse transcription-PCR assay using plasmid constructs containing specific sequences

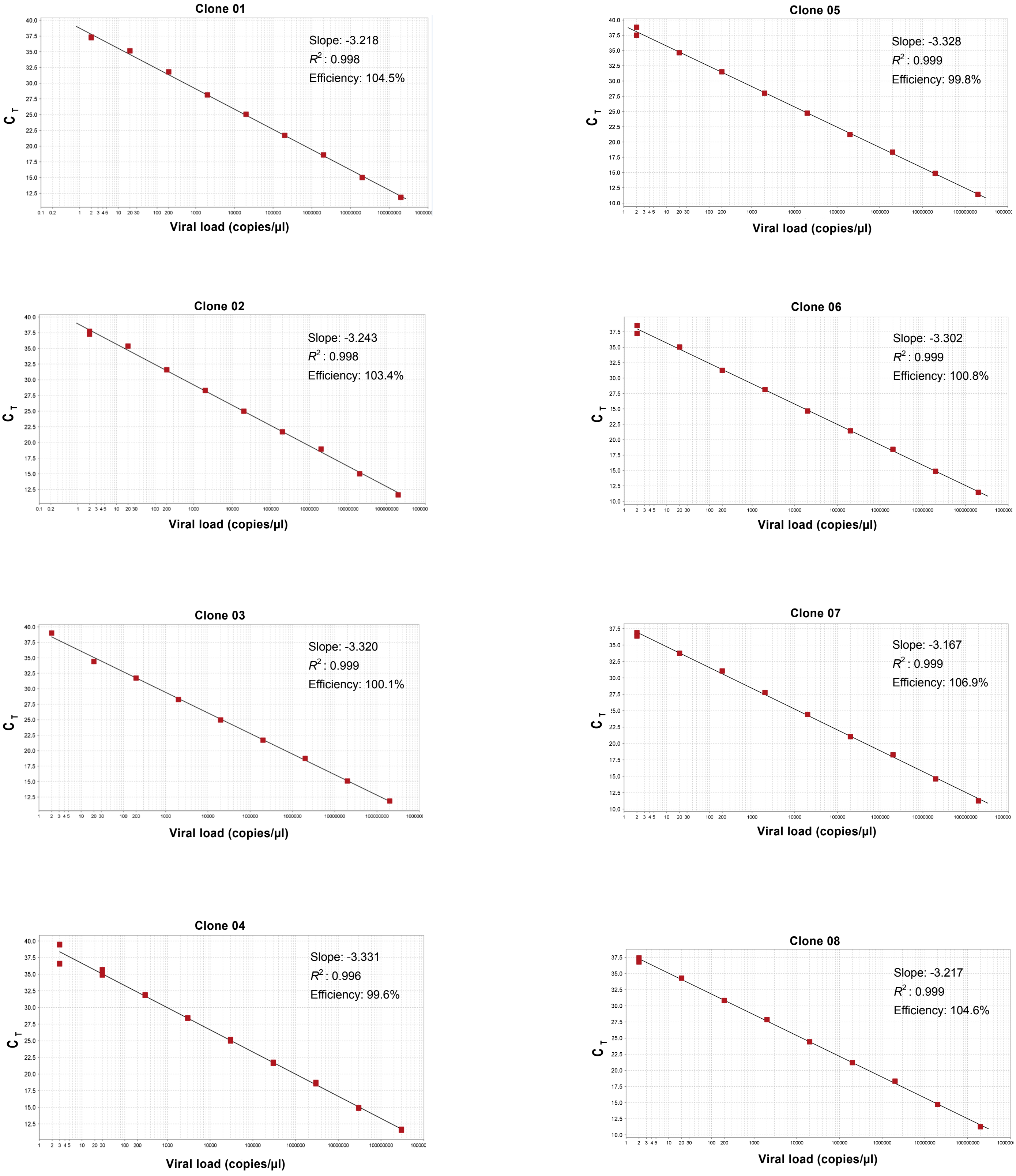

Supplementary Figure 1 (continued)

Performance of a Taqman Assay for Improved Detection and Quantification of Human Rhinovirus Viral Load

Kim Tien Ng, Jack Bee Chook, Xiang Yong Oong, Yoke Fun Chan, Kok Gan Chan, Nik Sherina Hanafi, Yong Kek Pang, Adeeba Kamarulzaman, and Kok Keng Tee

Standard curves of the real-time reverse transcription-PCR assay using plasmid constructs containing specific sequences

Clone 09

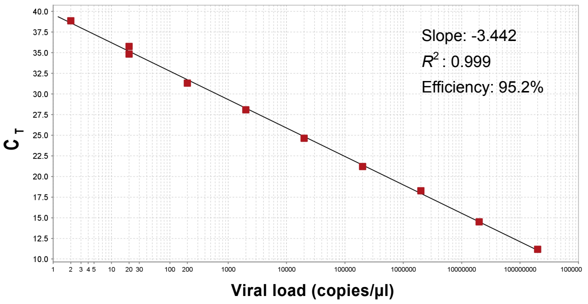

Clone 13

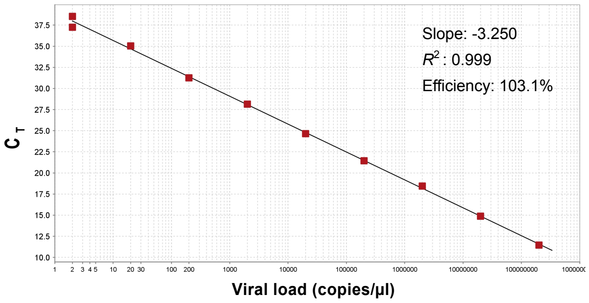

Clone 10

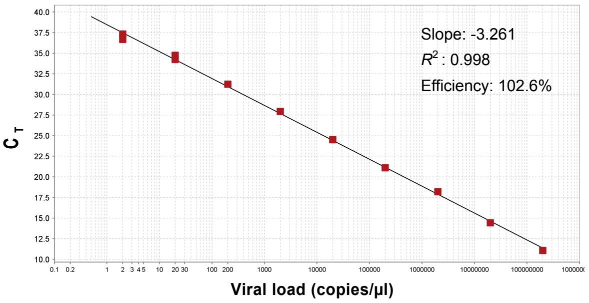

Clone 14

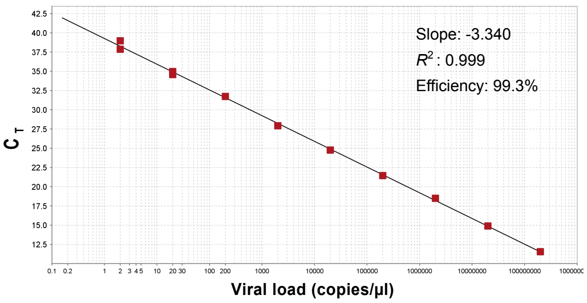

Clone 11

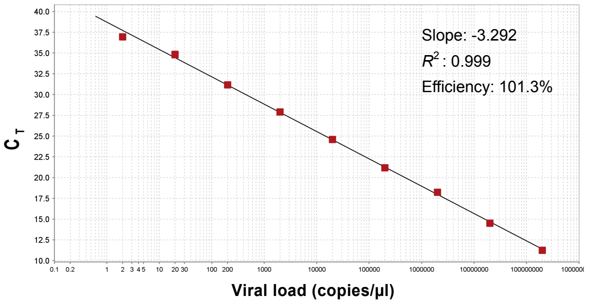

Clone 15

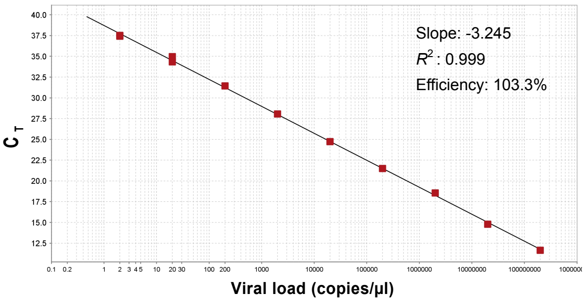

Clone 12

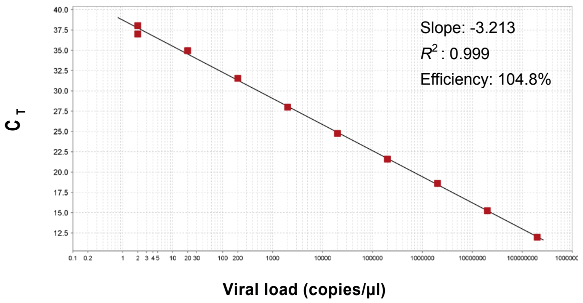

Clone 16

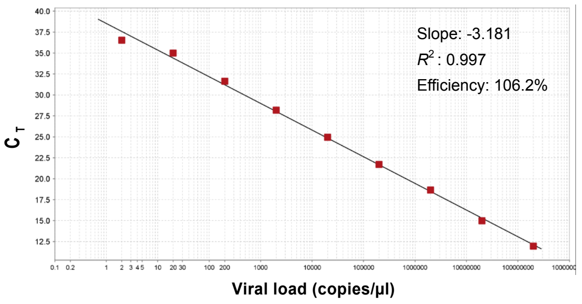

Supplementary Table 3

Performance of a Taqman Assay for Improved Detection and Quantification of Human Rhinovirus Viral Load

Kim Tien Ng, Jack Bee Chook, Xiang Yong Oong, Yoke Fun Chan, Kok Gan Chan, Nik Sherina Hanafi, Yong Kek Pang, Adeeba Kamarulzaman, and Kok Keng Tee

Supplementary Table 3A: The mean cycle threshold (C_T) values of the serially diluted plasmid constructs

Clone	Mean C_T values								
ID	10^8	10^7	10^6	10^5	10^4	10^3	10^2	10^1	10^0
01	11.85	15.02	18.59	21.70	25.07	28.14	31.82	35.15	37.34
02	11.66	15.19	18.95	21.43	24.99	28.32	31.60	35.39	37.27
03	11.86	15.10	18.74	21.70	24.98	28.28	31.75	34.43	39.01
04	11.45	14.80	18.34	21.37	24.86	28.22	31.55	34.22	37.85
05	11.44	14.86	18.35	21.22	24.74	27.99	31.52	34.64	38.81
06	11.45	14.87	18.44	21.44	24.66	28.15	31.26	35.03	37.89
07	11.26	14.60	18.26	21.05	24.44	27.76	31.06	33.75	36.89
08	11.27	14.72	18.32	21.20	24.42	27.86	30.82	34.29	37.11
09	11.18	14.50	18.28	21.20	24.64	28.09	31.31	34.83	35.76
10	11.08	14.44	18.59	21.11	24.51	27.94	31.26	34.74	36.66
11	11.25	14.49	18.23	21.19	24.59	27.91	31.16	34.80	36.95
12	11.97	15.24	18.60	21.59	24.76	28.01	31.55	34.97	38.04
13	11.33	14.75	18.31	21.33	24.54	27.90	31.43	34.38	37.18
14	11.85	14.96	18.50	21.43	24.78	27.93	31.72	34.97	37.86
15	11.66	14.78	18.56	21.49	24.72	28.05	31.44	34.33	37.53
16	11.96	14.98	18.66	21.71	24.94	28.19	31.64	35.01	36.54

Supplementary Table 3B: The PCR efficiency (*E*), coefficient of determination (R^2) and dynamic range of the plasmid constructs

Clone	*E* (%)	R^2	Dynamic range
**01**	104.5	0.998	10^8 to 10^0
**02**	103.4	0.998	10^8 to 10^0
**03**	100.1	0.999	10^8 to 10^0
**04**	99.6	0.996	10^8 to 10^0
**05**	99.8	0.999	10^8 to 10^0
**06**	100.8	0.999	10^8 to 10^0
**07**	106.9	0.999	10^8 to 10^0
**08**	104.6	0.999	10^8 to 10^0
**09**	95.2	0.999	10^8 to 10^0
**10**	102.6	0.998	10^8 to 10^0
**11**	101.3	0.999	10^8 to 10^0
**12**	104.8	0.999	10^8 to 10^0
**13**	103.1	0.999	10^8 to 10^0
**14**	99.3	0.999	10^8 to 10^0
**15**	103.3	0.999	10^8 to 10^0
**16**	106.2	0.997	10^8 to 10^0

Supplementary Table 4

Performance of a Taqman Assay for Improved Detection and Quantification of Human Rhinovirus Viral Load

Kim Tien Ng, Jack Bee Chook, Xiang Yong Oong, Yoke Fun Chan, Kok Gan Chan, Nik Sherina Hanafi, Yong Kek Pang, Adeeba Kamarulzaman, and Kok Keng Tee

Comparison between newly developed assay and previous approaches

Selected examples	Technical problems / weaknesses	Newly developed assay
a) Schibler et. al, 2012. Journal of Clinical Microbiology 50(9): 2868-2872		
b) Sachs et. al, 2011. Journal of Virological Methods 171:212-218	a) Random hexamer was used in cDNA synthesis. Such approach may over-estimate the viral load.	a) Specific primer targeting 5′-UTR of human rhinovirus will be used.
c) Lu et. al, 2008. Journal of Clinical Microbiology 46(2): 533-539	b) Due to the nature of the experiment ("two-step" RT-PCR), there is risk of contamination	b) One-step RT-PCR approach will be employed to minimize the risk of contamination.
d) Esposito et. al, 2014. European Journal of Clinical Microbiology & Infectious Diseases 33: 41-48		
e) Do et. al, 2010. Journal of Molecular Diagnostics 12(1): 102-108	Assay was tested on HRV-A and HRV-B, but not HRV-C	Primer set will be tested on HRV-A, HRV-B and HRV-C. Plasmids for each HRV species will be constructed.
f) Kotla et. al, 2009. Journal of Virological Methods 157(1): 32-39	Plasmid was constructed based on one of rhinovirus serotypes (HRV-A14). The primer set and probe were not tested for other human rhinoviruses (and their serotypes).	
